# The relationship between mode of delivery and Attention Deficit Hyperactivity Disorder: a meta-analysis and systematic review

**DOI:** 10.7717/peerj.20603

**Published:** 2026-01-16

**Authors:** Jiali Wei, Zehao Zheng, Yao Zheng, Kaishan Hou, Xue Pan, Xinyu Li, Yue Qiu, Mei Han

**Affiliations:** 1 Beijing University of Chinese Medicine, Beijing, China; 2 Changzhi Medical College, Shanxi, Shanxi, China; 3 Beijing University of Chinese Medicine DongFang College, Hebei, China; 4 The Third Affiliated Hospital of Beijing University of Chinese Medicine, Beijing, China; 5 Centre for Evidence-Based Medicine, Beijing University of Chinese Medicine, Beijing, China

**Keywords:** Childhood attention deficit hyperactivity disorder, Caesarean section, Meta-analysis, Vaginal delivery, Neurodevelopment

## Abstract

**Background:**

Attention Deficit Hyperactivity Disorder (ADHD) is a prevalent neurodevelopmental disorder in children, with an etiology that remains incompletely understood. Recent studies have suggested that the mode of delivery, particularly cesarean section (C-section), may be associated with an increased risk of ADHD. This study aims to examine whether children born *via* C-section are at increased risk of developing ADHD using both cohort and case-control data.

**Methods:**

We included observational studies (case-control or cohort) that examined the association between mode of delivery and ADHD in children. Inclusion criteria were: (a) ADHD diagnosed using standardized tools; (b) assessment of the relationship between C-section and ADHD; (c) availability of effect estimates (ORs with 95% CIs). Exclusion criteria were: (a) duplicate or overlapping data; (b) unavailable full text; (c) no extractable effect estimates. Literature searches were conducted in PubMed, Web of Science, CNKI, Wanfang, and VIP. Data were extracted using Excel and analyzed in R Studio with the meta, metabias, and metainf packages to pool the adjusted odds ratios (ORs) for the association between C-section and the occurrence of ADHD. Study quality was assessed using the Newcastle-Ottawa Scale (NOS).

**Results:**

A total of 14 studies were included, consisting of six case-control and eight cohort studies across 10 countries in Asia, Europe, the Americas, and Australia. ADHD diagnoses were based on DSM-IV or ICD-10. Most studies adjusted for key confounders such as maternal age (57.14%), child’s sex (50.00%), and gestational factors (35.71%). The pooled ORs were 1.44 (95% CI [1.04–1.25]) for case-control studies and 1.12 (95% CI [1.10–1.15]) for cohort studies. All studies scored ≥ 7 on the NOS.

**Conclusions:**

This meta-analysis suggests that C-section is associated with a moderately increased risk of ADHD in children. Both elective and emergency C-sections showed similar effects. However, limitations such as study heterogeneity, potential publication bias, and lack of genetic or biological mechanism data are to be acknowledged. Further research is needed to clarify causality and explore underlying mechanisms.

## Introduction

Attention-deficit/hyperactivity disorder (ADHD) constitutes a prevalent neurodevelopmental phenotype characterized by executive dysfunction across attentional, inhibitory, and motor control domains. The disorder exhibits substantial heritability and involves dysregulation of prefrontal-striatal neural circuits. Clinical manifestations typically emerge during early childhood, exhibiting dimensional rather than categorical expression patterns that persist across developmental trajectories into adulthood, thereby conferring significant functional impairment across academic, occupational, and psychosocial domains (Baumann et al., 2021). International epidemiological studies demonstrate that ADHD impacts roughly 7.2% of the pediatric population worldwide (Wolraich et al., 2019). United States National Survey data reveals diagnostic frequencies of 9.4% within the one-to-two-year age demographic in America (Wolraich et al., 2019). Contemporary meta-analytic evidence indicates ADHD occurrence rates of 6.26% among Chinese youth and adolescent populations (Shah et al., 2020).

ADHD represents a complex neurodevelopmental disorder with multifaceted pathological mechanisms involving genetic factors, environmental influences, epigenetics, and structural and functional brain abnormalities. Genetic factors play a crucial role in ADHD susceptibility, while the interaction between genes and environmental risk factors is considered key to its pathogenesis. Environmental factors include adverse influences during pregnancy and early life, which affect gene expression through epigenetic mechanisms and subsequently influence neurodevelopment (Zheng & Chen, 2018). ADHD patients exhibit widespread structural and functional brain abnormalities, particularly in the frontal lobe, basal ganglia, and cerebellum, regions associated with attention control, executive function, and impulse regulation. Functional connectivity and neurotransmission abnormalities in these brain regions constitute the primary pathophysiological foundation of ADHD (Da Silva et al., 2023; Shen & Zhou, 2024). Recent neuroimaging studies have revealed abnormalities in multiple neurotransmitter systems in ADHD patients, such as dopamine and norepinephrine systems, which play crucial roles in regulating attention and impulsive behavior. Furthermore, emerging “omics” research, including genomics and epigenetics, is unveiling the multifactorial pathological mechanisms of ADHD, providing possibilities for precision treatment (Da Silva et al., 2023).

Given that early life environmental factors significantly influence neurodevelopment and ADHD pathogenesis, the circumstances surrounding birth may constitute a critical period that shapes subsequent neurological outcomes. The mode of delivery represents one of the earliest environmental exposures that could potentially impact the developing nervous system through various biological pathways. Childbirth is the process by which a fetus is delivered from the mother’s body, signifying the start of an individual’s independent life (Supporting Healthy and Normal Physiologic Childbirth, 2013). Cesarean section (C-section) is a vital medical procedure used to protect the lives of both mother and baby when vaginal delivery is difficult or when specific pregnancy complications arise (Yang, 2021). However, elective C-sections performed without medical justification carry significant health risks for both mothers and infants—including a higher likelihood of hemorrhage, anesthesia-related complications, and surgical site infections from inadequate wound care—all of which can compromise maternal well-being (Mylonas & Friese, 2015). Moreover, extensive research has established associations between surgical delivery and elevated rates of immunological dysfunction, metabolic dysregulation, and altered neurodevelopmental trajectories across human and animal populations (Morais et al., 2021; Morais et al., 2020). Contemporary investigations utilizing cesarean-delivered murine models have revealed early developmental impairments in social behavioral repertoires and maternal attachment formation, alongside adult-onset cognitive deficits specifically affecting novel stimulus processing (Morais et al., 2021; Morais et al., 2020). Such experimental evidence emphasizes the potential neurodevelopmental ramifications associated with surgical delivery modalities.

Emerging evidence from large-scale human studies has begun to substantiate these concerns regarding C-section and neurodevelopmental outcomes. Several comprehensive cohort studies and meta-analyses have investigated the association between C-section and various neurodevelopmental disorders. Studies published in 2015 (Curran et al., 2015), 2019 (Zhang et al., 2019), 2024 (Chen et al., 2024) similarly reported a 10% to 30% increased risk of disorders such as Autism Spectrum Disorder (ASD) and ADHD associated with C-section. These findings from extensive human populations complement the mechanistic insights derived from animal models, suggesting that the relationship between delivery mode and neurodevelopmental outcomes extends beyond experimental settings to real-world clinical scenarios.

The potential biological mechanisms linking mode of delivery to ADHD development involve multiple pathways. Vaginal delivery exposes newborns to maternal microbiota, establishing initial gut colonization that influences the gut-brain axis and neurodevelopment (Liu et al., 2021). Conversely, C-section bypasses this microbial exposure, potentially altering early immune system programming and neuroinflammation pathways (Xu et al., 2023). Additionally, the hormonal cascade during natural labor, including oxytocin and stress hormones, plays crucial roles in brain maturation and behavioral regulation (Amikam et al., 2024). The absence of these physiological processes in C-section may contribute to altered neurodevelopmental trajectories, particularly affecting the dopaminergic and noradrenergic systems implicated in ADHD pathophysiology (Zhao et al., 2021).

The increase in global C-section rates is related to population growth in healthcare institutions (resulting in 66.5% of global growth) and the rising proportion of C-sections within healthcare institutions (resulting in 33.5% of global growth) (Delport, 2019). Globally, 60% of countries have C-section rates reaching 15%, with nearly 30% below 10% (Delport, 2019). Despite this guideline, cesarean deliveries continue to rise, notably in middle- and high-income nations (Kenkel, 2021). In China, according to the latest Lancet report in 2021, the C-section rate surged from 28.8% in 2008 to 34.9% in 2014 and further to 44.1% in 2020, marking the highest rate among Asian countries (Qiao et al., 2021; National Center for Quality Control of Obstetrics and Gynaecology & Chinese Medical Association Perinatal Medicine Branch, 2024). An analysis of extensive medical records revealed that the primary driver behind this increase is the use of non-clinical maternal risk factors, instead relying on subjective criteria such as unstable fetal status (Barber et al., 2011).

Considering the elevated C-section rates globally and the emerging evidence linking delivery mode to neurodevelopmental outcomes, investigating the potential association between C-section and childhood ADHD becomes increasingly important. This systematic review and meta-analysis aims to synthesize available evidence from case-control and cohort studies to determine whether mode of delivery influences ADHD risk in children. Notably, there is a paucity of studies specifically examining this association in Chinese populations, despite China having one of the highest C-section rates globally at 44.1% (Qiao et al., 2021; National Center for Quality Control of Obstetrics and Gynaecology & Chinese Medical Association Perinatal Medicine Branch, 2024). By incorporating comprehensive subgroup analyses based on study design, geographic region, and methodological quality, this study seeks to provide robust evidence that may inform clinical practice and guide efforts to reduce non-medically indicated cesarean deliveries.

## Methods

The protocol for this systematic review and meta-analysis was registered on INPLASY (Registration number: INPLASY202470124) and is available in full at https://doi.org/10.37766/inplasy2024.7.0124.

### Search strategy

This meta-analysis was conducted in accordance with the Preferred Reporting Items for Systematic Reviews and Meta-Analyses (PRISMA) and the Meta-analysis of Observational Studies in Epidemiology (MOOSE) guidelines. A comprehensive literature search was independently performed by two reviewers (JW and ZZ) across the following electronic databases: PubMed, the Cochrane Library, Web of Science, China National Knowledge Infrastructure (CNKI), VIP (China Science and Technology Journal Database), and Wanfang Data. Both English- and Chinese-language publications up to July 2024 were considered for inclusion. Any discrepancies in study selection or data extraction were resolved by consensus, with a third reviewer (MH) consulted when necessary. The detailed search strategy is outlined in Table S1.

### Inclusion and exclusion criteria

Inclusion criteria: (a) Studies involving participants diagnosed with ADHD using standardized diagnostic criteria; (b) Studies investigating the association between C-section and ADHD; (c) Observational study designs, including case-control and cohort studies. Exclusion criteria: (a) Studies based on overlapping datasets to avoid participant duplication; (b) Studies lacking available odds ratios (ORs) and 95% confidence intervals (CIs); (c) Studies without access to full text; (d) Redundant publications or duplicated reports of the same data.

### Literature screening and data extraction

Initial screening was conducted using NoteExpress software. Two reviewers independently screened titles and abstracts based on predefined inclusion and exclusion criteria. In cases of disagreement, a third reviewer adjudicated. Full-text articles of potentially eligible studies were downloaded and re-evaluated independently by the same two reviewers. Data were then extracted independently, including general study information, study design, sample size, outcome measures (adjusted ORs and 95% CIs), adjustment variables, and quality assessment indicators for both case-control and cohort studies. After extraction, data consistency was cross-verified, with discrepancies resolved through discussion or third-party arbitration.

### Quality assessment

The methodological quality of included case-control and cohort studies was assessed using the Newcastle–Ottawa Scale (NOS) (Wells et al., 2014), which evaluates three domains: selection of study participants, comparability of groups, and ascertainment of either exposure (for case-control studies) or outcome (for cohort studies). Assessment results were presented graphically.

### Subgroup analysis

Subgroup analyses were conducted based on the type of C-section, distinguishing between elective and emergency procedures. Elective C-sections are typically planned due to maternal request or combined maternal-fetal considerations, such as severe tokophobia (Almqvist & Rejnö, 2013). Emergency C-sections are performed in response to acute complications, such as fetal distress or onset of labor prior to a scheduled elective procedure, where vaginal delivery is deemed unfeasible (Almqvist & Rejnö, 2013). Differentiating between these types allows for a more nuanced understanding of their respective impacts on maternal and neonatal outcomes, and may inform targeted clinical interventions.

### Statistical analyses

Meta-analyses were conducted using the *meta* package in R Studio. Effect sizes from included case-control and cohort studies were expressed as odds ratios (ORs) with corresponding 95% confidence intervals (CIs). A fixed-effects model was applied when heterogeneity was low (I^2^ < 50%), while a random-effects model was employed for substantial heterogeneity (I^2^ > 50%). Given the diversity in ethnicity, geographical region, and sample size across the studies, a random-effects model was used throughout. Sensitivity analyses were performed using the *metainf* package in R Studio to identify potential sources of heterogeneity.

### Publication bias

Publication bias was assessed using funnel plots and the *metabias* function in R Studio to visually and statistically evaluate asymmetry among the included studies.

## Results

### Study selection

**Figure 1:**
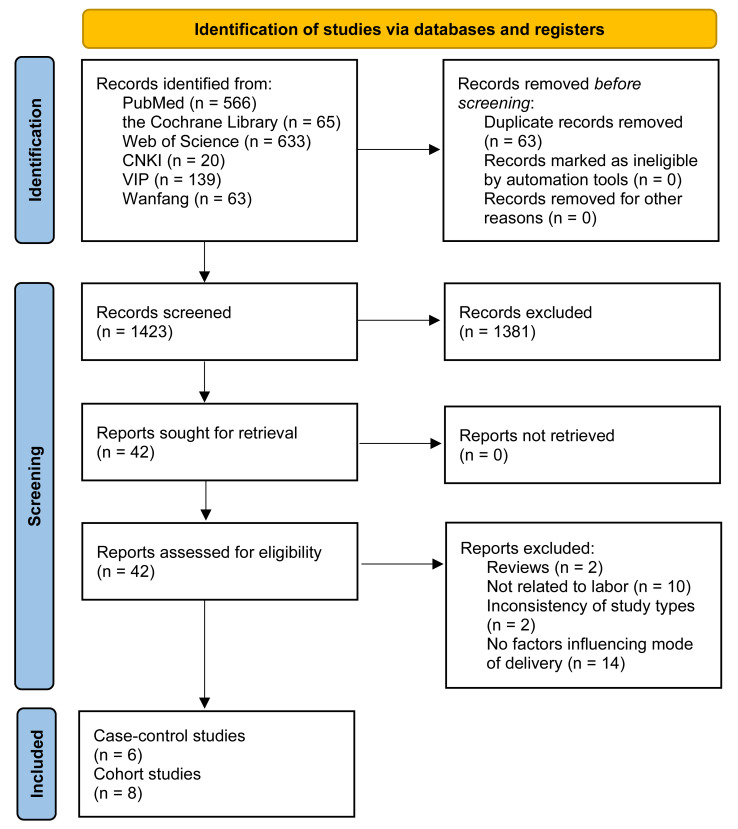
PRISMA flowchart of literature screening and selection process. The systematic literature search and selection process following PRISMA guidelines. The diagram shows the number of records identified from multiple databases, screening procedures, and exclusion criteria applied to arrive at the final studies included in the meta-analysis.

A total of 1,486 records were retrieved through database searches. After removing duplicates and screening titles and abstracts, 1,423 studies remained. Of these, 1,381 were excluded for being laboratory research, animal studies, or systematic reviews. The remaining 42 full-text articles were assessed for eligibility. Twenty-five studies were excluded for the following reasons: two were reviews, 10 were irrelevant to childbirth, two did not meet the study design criteria, and 14 lacked variables related to mode of delivery. Ultimately, six case-control studies and eight cohort studies met the inclusion criteria. The study selection process is detailed in the PRISMA flowchart (Fig. 1).

### Characteristics of the included studies

The six case-control studies were conducted in Iran, Norway, Finland, and China, spanning Europe, Asia, and Australia. These studies were published between 2011 and 2024. ADHD diagnoses were based on established criteria, including the DSM-IV and ICD-10. Most studies adjusted for confounding factors such as gender, maternal health, pregnancy characteristics, and delivery conditions. Study characteristics are summarized in Table 1.

The eight included cohort studies were prospective in design and conducted in Denmark, China, Sweden, the United Kingdom, Brazil, and Germany, covering Asia, Europe, and the Americas. Publication years ranged from 2013 to 2023. ADHD was diagnosed using the DSM-IV, ICD-10, or the Strengths and Difficulties Questionnaire (SDQ). Half of the studies reported adjusting for key confounders, including gender and maternal health. Study characteristics are presented in Table 1.

### Quality assessment

The included studies demonstrated rigorous control of potential confounders, essential for ensuring the validity of observational findings. Commonly adjusted variables included maternal age (57.14% of studies), child’s gender (50.00%), delivery mode (35.71%), exposure to tobacco (28.57%) or alcohol (21.43%), parity (28.57%), marital status (21.43%), and gestational age (21.43%). All case-control studies achieved a NOS score of ≥8, indicating high methodological quality. Detailed scoring is provided in Table S2. NOS scores for cohort studies ranged from 7 to 9, reflecting generally strong study quality. Full scoring details are available in Table S3.

### Meta-analysis of the association between C-section and ADHD

#### Case-control studies

**Figure 2:**
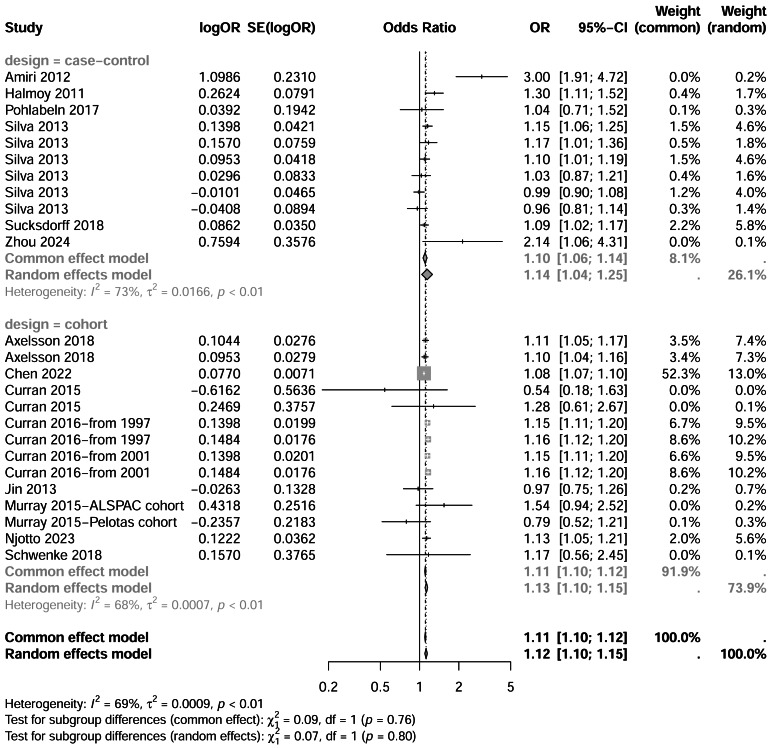
Forest plot of the association between cesarean section delivery and ADHD risk. Forest plot showing odds ratios (OR) and 95% confidence intervals for the association between cesarean section delivery and ADHD risk compared to vaginal delivery. Studies are stratified by study design (case-control *vs.* cohort studies). Diamond symbols represent pooled estimates using common effect and random effects models. Heterogeneity statistics (I^2^) and *p*-values are provided for each subgroup. Note: Amiri et al. (2012); Halmøy et al. (2012); Pohlabeln et al. (2017); Silva et al. (2014); Sucksdorff et al. (2018); Zhou et al. (2024); Axelsson et al. (2019); Chen et al. (2023); Curran et al. (2015); Curran et al. (2016a); Curran et al. (2016b); Jin et al. (2014); Murray et al. (2016); Njotto et al. (2023); Schwenke et al. (2018).

Six case-control studies were included. Silva et al. (2014) reported multiple adjusted ORs, all of which were incorporated. Significant heterogeneity was observed (I^2^ = 73%, *p* < 0.01), and a random-effects model was applied. The pooled OR for ADHD associated with C-section was 1.44 (95% CI [1.04–1.25]), indicating a statistically significant association. The forest plot is shown in Fig. 2.

#### Cohort studies

Eight cohort studies were analyzed. Axelsson et al. (2019) and Curran et al. (2015) provided multiple adjusted ORs, while Curran et al. (2016a), Curran et al. (2016b) and Murray et al. (2016) used multiple databases; all data were included. Heterogeneity was moderate (I^2^ = 69%, *p* < 0.01), and a random-effects model was used. The pooled OR was 1.12 (95% CI [1.10–1.15]), suggesting that children born *via* C-section had a modest but statistically significant increased risk of ADHD. See forest plot in Fig. 2.

### Subgroup analysis by C-section type

**Figure 3:**
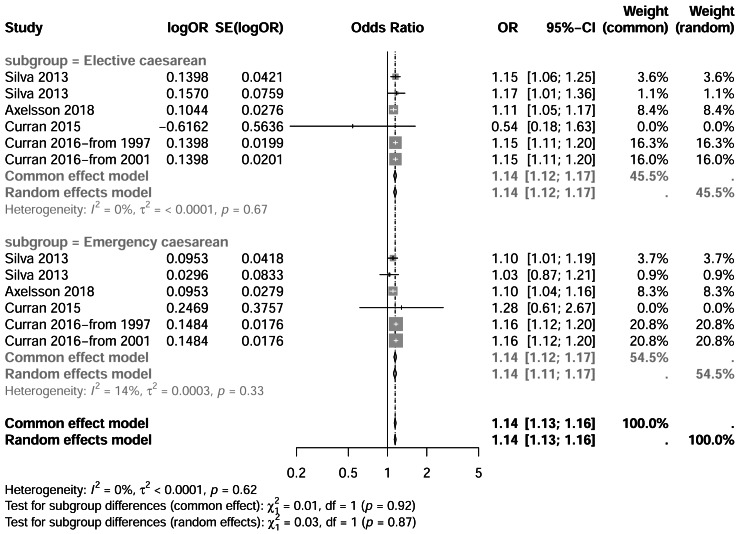
Forest plot comparing elective *versus* emergency cesarean section and ADHD risk. Forest plot displaying odds ratios (OR) and 95% confidence intervals for the association between elective and emergency cesarean section deliveries and ADHD risk. Studies are stratified by cesarean section type. Diamond symbols show pooled estimates from common effect and random effects models. Tests for subgroup differences and heterogeneity statistics are presented. Note: Silva et al. (2014); Axelsson et al. (2019); Chen et al. (2023); Curran et al. (2015); Curran et al. (2016a); Curran et al. (2016b).

Subgroup analyses were performed by C-section type: elective *vs.* emergency. For elective C-sections, heterogeneity was minimal (I^2^ = 0%, *p* = 0.69), and a fixed-effects model yielded a pooled OR of 1.14 (95% CI [1.12–1.17]). Similarly, emergency C-sections showed no heterogeneity (I^2^ = 0%, *p* = 0.33), with a pooled OR of 1.14 (95% CI [1.13–1.16]). These comparable effect sizes suggest that both elective and emergency C-sections are associated with an increased risk of ADHD. Results are illustrated in Fig. 3.

**Table 1:** Characteristics of all the studies included in the meta-analysis. This table summarizes the key characteristics of all studies examining the association between perinatal factors and ADHD included in the systematic review and meta-analysis. Data presented includes study design, population demographics, sample sizes, ADHD diagnostic criteria, and confounding variables adjusted for in each study analysis.

Study	Country	Data source	Period	Age of ADHD measurement (years)	Sample size (Case/Control)	Male/Female	ADHD measurement	Adjusted factors	Study type
Amiri et al. (2012)	Iran	Tabriz University of Medical Sciences	2009	Not specifically provided, mentioned as school-aged children	164/166	NA	ADHD Rating Scale—Parent Version; Schedule for Affective Disorders and Schizophrenia for School-Aged Children	Maternal somatic diseases, maternal psychiatric disorders, alcohol and cigarette exposure, mode of delivery	Case-control study
Halmøy et al. (2012)	Norway	The Medical Birth Registry of Norway	1997–2005	Born between 1967–1987, adults at the time of the study (specific ages not provided)	2,323/1,170,073	NA	By the referring clinicians	Maternal age, parity, time period of birth (5-year categories), maternal educational level, maternal marital status, and gender	Case-control study
Pohlabeln et al. (2017)	Europe	The European IDEFICS study	2007–2010	2–11.9 y	192/15,385	7,884/7,693	IDEFICS parental questionnaire, IDEFICS medical questionnaire	Cigarette exposure, gestational hypertension, neonatal respiratory disorders, infections during the first 4 weeks after birth	Case-control study
Silva et al. (2014)	Western Australia	MODDS, Midwives Notification System (MNS)	Aug 2003–2007	Not specifically provided, referred to as children and adolescents	12,991/30,071	333,221/9,841	DSM-IV and ICD-10	Maternal age, marital status, firstborn, cigarette exposure, pregnancy complications, maternal urinary tract infection, preeclampsia, premature rupture of membranes, fetal distress, *etc.*	Case-control study
Sucksdorff et al. (2018)	Finland	Finnish Hospital Discharge Register, Medical Birth Register, Central Population Register	Jan 1991–Dec 2005	Not specifically provided, mentioned as children	10,409/39,124	NA	DSM-IV and ICD-10	Gestational age, weight for gestational age, maternal age, maternal socioeconomic status, alcohol and cigarette exposure, substance abuse, psychiatric disorders	Case-control study
Zhou et al. (2024)	China	Dept of Child Healthcare, Children’s Hospital, Capital Institute of Pediatrics	May 2021–Aug 2023	6–8 y	272/117	206/66	DREAM-C	Mode of delivery, family relationship, premature screen exposure, family history of ADHD	Case-control study
Axelsson et al. (2019)	Denmark	Register linkage via national ID numbers	1997–2010	NA	117,865/553,727	NA	ICD-10	Family history of ADHD, childhood antibiotics use, mode of delivery, maternal age at birth, parental age difference, parental education, maternal marital status, cigarette exposure, gender, Apgar score, CPAP/ventilator, asphyxia, parental epilepsy, preeclampsia, gestational diabetes, labor induction, maternal antibiotics, infections	Cohort study
Chen et al. (2023)	China	Taiwan Maternal & Child Health Database, Birth Certificate Registration	2004–2006	NA	676,608/1,208,093	981,929/902,772	ICD-10	Gender, maternal age, maternal neurodevelopmental disorders	Cohort study
Curran et al. (2015)	Sweden	Swedish national registers (Birth, Patient, Multi-generation)	1990–2008	>3	238,687/1,483,852	883,115/839,433	ICD-10	Gestational age, maternal BP, pre-eclampsia, cigarette exposure, firstborn, miscarriage, poverty, ethnicity, maternal age, education, depression, BMI, gender, urbanicity, single parent, paternal age, paternal education, unplanned pregnancy, maternal IBS	Cohort study
Curran et al. (2016a) and Curran et al. (2016b)	UK	Millennium Cohort Study (18,827 children)	2000–2002	9 months; 3, 5, 7, 11 y	2,815/10,247	9,364/3,698	SDQ	Gender, maternal age, gestational age, parental citizenship, SGA, LGA, Apgar, parity, smoking, welfare, education, income, parental depression, bipolar, non-affective disorder	Cohort study
Jin et al. (2014)	China	One million population frame, Zhabei District, Shanghai	Apr–May 2009	5–15 y	2,691/3,133	2,787/2,828	Clinical interviews by psychiatrists	Gender, household situation, income per capita, father’s education, mother’s education	Cohort study
Murray et al. (2016)	UK, Brazil	ALSPAC (UK); Pelotas (Brazil)	1991–1992	7.23 y	ALSPAC (*N* = 6,768); Pelotas (*N* = 3,508)	NA	SDQ and DSM-IV	LBW, SGA, HC, PTB	Cohort study
Njotto et al. (2023)	Sweden	Swedish nationwide registries	2006–2016	11 y	90,695/392,800	249,075/234,384	ICD-10	Mode of delivery, maternal age, BMI, cigarette exposure, parity, family situation, gender, gestational age, Apgar, size for gestational age	Cohort study
Schwenke et al. (2018)	Germany	Erlangen University Hospital	1996–1999	10–13 y	112/443	NA	Mother’s statement	Maternal age, maternal weight gain, pre-pregnancy weight, end-pregnancy weight, maternal height, education, pregnancy number, alcohol, cigarette exposure, mode of delivery, gestational week, birthweight, umbilical artery blood values, Apgar (5, 10 min), breastfeeding	Cohort study

**Notes.**

NA not available ICD-10 International Classification of Diseases 10th revision SDQ the Strengths and Difficulties Questionnaire DSM-IV Development and Well-Being Assessment IDEFICS Identification and prevention of dietary- and lifestyle-induced health effects in children and infants ALSPAC Avon Longitudinal Study of Parents and Children MODDS the Monitoring of Drugs of Dependence System MNS Midwives Notification System DREAM-C Diagnostic Receptive and Expressive Assessment of Mandarin-Comprehensive CPAP Continuous Postive Airway Pressure BMI body mass index Gestational Age (SGA, AGA, LGA) SGA, Small for Gestational Age AGA Appropriate for Gestational Age LGA Large for Gestational Age IBS irritable bowel syndrome LBW low birth weight HC small head circumference PTB preterm birth

### Publication bias

**Figure 4:**
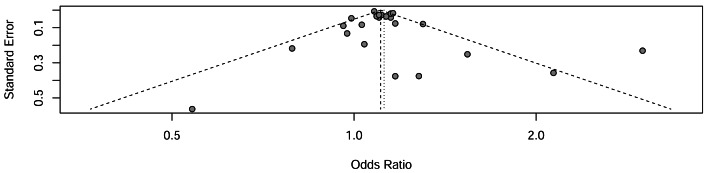
Funnel plot for assessment of publication bias. Funnel plot examining potential publication bias in the meta-analysis. The *x*-axis represents the odds ratio and the *y*-axis shows the standard error. Symmetrical distribution of studies around the pooled estimate suggests absence of significant publication bias.

Funnel plot analysis revealed symmetrical distributions, suggesting minimal publication bias among the included studies. Results are presented in Fig. 4.

### Sensitivity analysis

Sensitivity analyses demonstrated that the pooled results remained statistically significant (*p* < 0.01) even after excluding any single study, indicating the robustness and reliability of the findings. Details can be found in Table S4.

## Discussion

### Main findings

This meta-analysis of case-control and cohort studies demonstrates a statistically significant association between C-section and an increased risk of ADHD in children, with an overall risk increase of approximately 14%. Both elective and emergency C-sections were associated with elevated ADHD risk, with no significant difference observed between the two modes of delivery.

### Elective *vs.* emergency C-sections

Our subgroup analyses revealed comparable associations between ADHD and both elective and emergency C-sections. However, the potential mechanisms underlying these associations may differ. Emergency C-sections are typically performed in response to obstetric complications such as fetal distress, dystocia, or maternal indications, which themselves may contribute to neurodevelopmental outcomes (Almqvist & Rejnö, 2013). A large cohort study published in 2021 (Zhang et al., 2021) reported that the association between emergency C-section and ADHD was substantially attenuated after adjusting for perinatal complications, suggesting that elective C-sections may have a more direct influence on ADHD risk.

A growing body of evidence from both human and animal studies supports the notion that C-section can influence neurobehavioral development (Huang et al., 2019). For instance, cesarean-born rodents exhibit deficits in social cue recognition (Morais et al., 2021; Morais et al., 2020). Vaginal delivery provides mechanical and hormonal stimuli that facilitate critical neonatal physiological adaptations, including stress hormone release, thermoregulation, and metabolic transitions (Kiilerich et al., 2021; Wampach et al., 2018; Schuller et al., 2012; Kuwabara et al., 1987). A Danish cohort study found that neonates delivered *via* C-section, particularly elective procedures, exhibited lower levels of inflammatory and stress markers, as well as reduced concentrations of growth factors compared to those delivered vaginally (Kiilerich et al., 2021).

Although our meta-analysis did not detect a significant difference in ADHD incidence between elective and emergency C-sections, the underlying biological consequences may diverge. Elective procedures, being planned and devoid of the physiological stress associated with labor, may deprive neonates of key stress-related hormonal surges important for neurodevelopment (Zhang et al., 2021; Kuwabara et al., 1987). In contrast, emergency C-sections, often accompanied by acute maternal-fetal distress, may exert complex, context-dependent influences on brain development (Zhang et al., 2021).

### Potential mechanisms

Several biological mechanisms have been proposed to explain the association between C-section and altered neurodevelopmental outcomes. Among the most studied are dysregulation of the hypothalamic-pituitary-adrenal (HPA) axis, impaired oxytocin signaling, and disruptions to the early establishment of gut microbiota.

Vaginal delivery induces mechanical and hormonal stimuli that activate specific brain regions, such as the piriform cortex and caudate nucleus, promoting early-life stress responses and the functional maturation of the HPA axis (Kenkel et al., 2019). In contrast, cesarean-born neonates exhibit significantly lower levels of glucocorticoids at birth, which may hinder the development of stress-adaptive systems. Animal studies have demonstrated that inadequate glucocorticoid signaling can inhibit neurogenesis and synaptogenesis, particularly in the hippocampus, a critical region for learning and memory (Kenkel et al., 2019). Cesarean-delivered mice also show lower corticosterone levels at birth and later display cognitive deficits during adolescence (Huang et al., 2019). In human neonates, cortisol levels are highest following vaginal delivery, intermediate in emergency C-sections, and lowest in elective C-sections (Miller et al., 2005), suggesting a gradient of early stress exposure with potential implications for neurodevelopment.

In parallel, the oxytocin system—crucial for regulating social behavior, emotional bonding, and stress reactivity—is also influenced by delivery mode. Neonates born *via* vaginal delivery have been shown to exhibit higher circulating oxytocin levels compared to those delivered by C-section (Kuwabara et al., 1987; Kenkel et al., 2019; Green et al., 2021). Experimental studies further reveal that early oxytocin administration in cesarean-born mice not only improves social and anxiety-like behaviors but also enhances immune function (Morais et al., 2021; Morais et al., 2020), indicating a modulatory role of oxytocin in postnatal brain development.

Another plausible pathway involves the gut-brain axis. C-section significantly alters the initial colonization of the infant gut microbiota by interrupting vertical transmission of maternal microbial strains, thereby affecting the composition and function of the neonatal gut microbiome (Sherwin et al., 2019). These changes have been associated with long-term effects on cognition, emotional regulation, and social interaction (Neumann & Slattery, 2016; Wei et al., 2009; Codagnone et al., 2019; Dinan & Cryan, 2017; Yang et al., 2016). Moreover, psychological stress experienced during early development can further modify gut microbial communities, reinforcing the bidirectional relationship between gut health and brain function (Morais et al., 2021; Morais et al., 2020; Wampach et al., 2018; Xu et al., 2020a; Xu et al., 2020b). Intriguingly, oxytocin treatment in animal models not only modulated behavior but also partially restored gut microbial diversity in cesarean-born mice (Dangoor et al., 2021).

Collectively, these findings suggest a complex interplay between hormonal, neuroendocrine, and microbial systems in shaping neurodevelopmental trajectories following C-section. However, the precise causal pathways remain to be fully elucidated and warrant further investigation in longitudinal and mechanistic studies.

### Implications for neurodevelopmental disorders

Our findings align with previous research suggesting that C-section may confer increased risk for a broader spectrum of neurodevelopmental disorders. Prior meta-analyses have reported a 10–30% elevated risk of conditions such as autism spectrum disorder (ASD) and ADHD among cesarean-born individuals (Curran et al., 2015; Zhang et al., 2019; Chen et al., 2024). However, earlier studies often grouped neurodevelopmental outcomes together, lacking disorder-specific granularity. A 2020 meta-analysis focusing solely on ADHD concluded that the observed associations might have been overstated, in part due to limited population diversity and methodological inconsistencies (Xu et al., 2020a; Xu et al., 2020b).

In addition to ADHD, recent large-scale cohort studies have elucidated further links between C-section and early neurodevelopmental impairments. A study based on the Japan Environment and Children’s Study (JECS) reported that C-section was associated with an increased risk of neurodevelopmental disorders at age three, including motor delays, intellectual disabilities, and ASD. Interestingly, the strength of these associations varied by sex, underscoring the potential for sex-specific vulnerability to cesarean-associated neurodevelopmental disruption (Yoshida et al., 2023). Similarly, a prospective cohort study conducted in a Shanghai Jiao Tong University-affiliated hospital found a significant association between C-section and sensory integration dysfunction in preschool children—a neurodevelopmental condition affecting motor coordination, attention, and sensory processing (Zhao et al., 2021). These findings collectively suggest that C-section may influence multiple domains of early brain development, though the exact biological mechanisms remain to be fully elucidated.

Taken together, the evidence suggests that reducing unnecessary elective cesarean deliveries may contribute to lowering the population burden of ADHD and potentially other neurodevelopmental disorders.

### Strengths and limitations

This study has several strengths, including a comprehensive literature search across multiple databases, inclusion of both case-control and cohort designs, and representation of diverse populations across Western and Asian countries. Subgroup analyses by delivery type further enriched the interpretation.

Nevertheless, certain limitations must be acknowledged. First, as an observational meta-analysis, residual confounding cannot be fully excluded. Second, substantial heterogeneity was observed across studies, stemming from variability in sample sizes, ethnic backgrounds, diagnostic criteria, and covariate adjustment strategies. Third, the number of eligible studies remains limited, potentially affecting the robustness and generalizability of the findings. Additionally, outcomes were not stratified according to specific medical indications for cesarean delivery, but rather based solely on whether the procedure was elective or emergency; such a distinction may hinder the ability to isolate independent effects. Finally, differences in outcome assessment and follow-up periods may introduce bias and limit cross-study comparability.

## Conclusions

This meta-analysis provides evidence that C-section is associated with an increased risk of attention deficit hyperactivity disorder (ADHD) in children. The observed associations were consistent across both elective and emergency C-section. Although the underlying mechanisms may differ, the comparable effect sizes suggest that both types of C-section warrant attention in public health and clinical decision-making. From a preventive perspective, reducing the rate of non-medically indicated elective C-sections and optimizing the clinical criteria for emergency surgical interventions may contribute to lowering the population burden of ADHD. Further prospective and mechanistic studies are needed to clarify causal pathways and inform early intervention strategies.

##  Supplemental Information

10.7717/peerj.20603/supp-1 Supplemental Information 1 PRISMA 2020 Checklist This checklist documents adherence to the PRISMA 2020 reporting guidelines for the systematic review and meta-analysis examining the association between cesarean section delivery and childhood ADHD risk.

10.7717/peerj.20603/supp-2 Supplemental Information 2 Target Audience This section describes the intended readership for the manuscript, encompassing healthcare professionals, researchers, and policy makers across multiple disciplines who would benefit from understanding the relationship between delivery mode and ADHD risk. The diverse audience reflects the interdisciplinary nature of perinatal and neurodevelopmental research.

10.7717/peerj.20603/supp-3 Supplemental Information 3 Search strategy for systematic literature review Detailed search terms and strategies used across multiple databases to identify relevant studies examining the association between mode of delivery and ADHD. Search terms included combinations of delivery method terminology and ADHD-related keywords in both English and Chinese languages.

10.7717/peerj.20603/supp-4 Supplemental Information 4 Quality assessment of case-control studies using Newcastle-Ottawa Scale Quality assessment results for case-control studies included in the meta-analysis using the Newcastle-Ottawa Quality Assessment Scale (NOS) checklist. The NOS evaluates study quality based on selection of study groups, comparability of groups, and ascertainment of exposure.

10.7717/peerj.20603/supp-5 Supplemental Information 5 Quality assessment of cohort studies using Newcastle-Ottawa Scale Quality assessment results for cohort studies included in the meta-analysis using the Newcastle-Ottawa Quality Assessment Scale (NOS) checklist. The NOS evaluates study quality based on selection of study groups, comparability of groups, and assessment of outcomes.

10.7717/peerj.20603/supp-6 Supplemental Information 6 Sensitivity analysis by sequential omission of individual studies Results of sensitivity analysis examining the robustness of pooled estimates by systematically excluding each study one at a time. The table shows odds ratios, confidence intervals, p-values, and heterogeneity statistics (*I*^2^) for each omission scenario to assess the influence of individual studies on overall results.

10.7717/peerj.20603/supp-7 Supplemental Information 7 Codebook

## Abbreviations

ADHD: Attention deficit hyperactivity disorder
C-section: Cesarean section
ICD-10: International Classification of Diseases 10th revision
SDQ: the Strengths and Difficulties Questionnaire
DSM-IV: Development and Well-Being Assessment
IDEFICS: Identification and prevention of dietary- and lifestyle-induced health effects in children and infants
ALSPAC: Avon Longitudinal Study of Parents and Children
MODDS: the Monitoring of Drugs of Dependence System
MNS: Midwives Notification System
DREAM-C: Diagnostic Receptive and Expressive Assessment of Mandarin-Comprehensive
CPAP: Continuous Postive Airway Pressure
BMI: body mass index

## Gestational Age (SGA, AGA, LGA)

SGA: Small for Gestational Age
AGA: Appropriate for Gestational Age
LGA: Large for Gestational Age
IBS: irritable bowel syndrome
LBW: low birth weight
HC: small head circumference
PTB: preterm birth

## Additional Information and Declarations

### Data Availability

The following information was supplied regarding data availability: This is a systematic review/meta-analysis.
