# Supplementary material for: The relationship between mode of delivery and Attention Deficit Hyperactivity Disorder: a meta-analysis and systematic review
Source: PeerJ. 2026 Jan 16;14:e20603. doi: 10.7717/peerj.20603 (PMC12814906; doi:10.7717/peerj.20603)
Supplement: Supplemental Information 2 — This section describes the intended readership for the manuscript, encompassing healthcare professionals, researchers, and policy makers across multiple disciplines who would benefit from understanding the relationship between delivery mode and ADHD risk. The diverse audience reflects the interdisciplinary nature of perinatal and neurodevelopmental research. [file peerj-14-20603-s002.docx]

This manuscript is intended for researchers, clinicians, and public health professionals with an interest in neurodevelopmental disorders, perinatal medicine, and epidemiology. Specifically, the audience includes:

1. Pediatricians and Child Psychiatrists: Professionals diagnosing and managing ADHD who seek to understand potential early-life risk factors influencing neurodevelopmental outcomes.
2. Obstetricians and Gynecologists: Healthcare providers involved in perinatal care, particularly those advising expectant mothers on delivery methods and associated risks.
3. Epidemiologists and Public Health Experts: Researchers analyzing trends in cesarean delivery rates and their broader implications on childhood health.
4. Neuroscientists and Developmental Psychologists: Experts exploring the biological and behavioral impacts of birth mode on cognitive and emotional development.
5. Healthcare Policy Makers: Individuals involved in formulating guidelines on childbirth practices and maternal-infant health policies.
