# Supplementary material for: The relationship between mode of delivery and Attention Deficit Hyperactivity Disorder: a meta-analysis and systematic review
Source: PeerJ. 2026 Jan 16;14:e20603. doi: 10.7717/peerj.20603 (PMC12814906; doi:10.7717/peerj.20603)
Supplement: Supplemental Information 3 — Detailed search terms and strategies used across multiple databases to identify relevant studies examining the association between mode of delivery and ADHD. Search terms included combinations of delivery method terminology and ADHD-related keywords in both English and Chinese languages. [file peerj-14-20603-s003.docx]

**Supplementary Table S1 Search strategy**

| Database | Search strategy |
| --- | --- |
| PubMed | (mode of delivery[Title/Abstract] OR method of delivery[Title/Abstract] OR delivery[Title/Abstract] OR birth delivery[Title/Abstract] OR caesarean birth[Title/Abstract] OR cesarean[Title/Abstract] OR cesarean section[Title/Abstract] OR Caesarean section[Title/Abstract] OR cesarean delivery[Title/Abstract]) AND ((ADHD[Title/Abstract] OR adhd[Title/Abstract] OR attention deficit hyperactivity disorder[Title/Abstract] OR child hyperkinetic syndrome[Title/Abstract] OR childhood hyperkinetic syndrome[Title/Abstract] OR infantile hyperkinetic syndrome[Title/Abstract])) |
| the Cochrane Library | mode of delivery OR method of delivery OR delivery OR birth delivery OR caesarean birth OR cesarean OR cesarean section OR Caesarean section OR cesarean delivery:ti,ab,kw ADHD OR adhd OR attention deficit hyperactivity disorder OR child hyperkinetic syndrome OR childhood hyperkinetic syndrome OR infantile hyperkinetic syndrome:ti,ab,kw |
| Web of Science | TS=(mode of delivery OR method of delivery OR delivery OR birth delivery OR caesarean birth OR cesarean OR cesarean section OR Caesarean section OR cesarean delivery ) AND TS= (ADHD OR adhd OR attention deficit hyperactivity disorder OR child hyperkinetic syndrome OR childhood hyperkinetic syndrome OR infantile hyperkinetic syndrome) |
| CNKI | SU=('cesarean delivery' OR 'cesarean section' OR 'caesarean delivery' OR 'caesarean section' OR 'C-section' OR 'delivery mode' OR 'mode of delivery') AND SU=('ADHD' OR 'attention deficit hyperactivity disorder' OR 'attention deficit disorder' OR 'hyperkinetic disorder' OR 'hyperkinetic syndrome' OR 'attention deficit hyperactivity disorder' OR 'childhood hyperactivity' OR 'hyperactive child syndrome' OR 'minimal brain dysfunction') |
| Wanfang | Topic: (cesarean delivery OR cesarean section OR caesarean delivery OR caesarean section OR C-section OR delivery OR delivery mode OR mode of delivery) AND Topic: (ADHD OR attention deficit hyperactivity disorder OR attention deficit disorder OR hyperkinetic disorder OR hyperkinetic syndrome OR childhood hyperactivity OR hyperactive child syndrome OR minimal brain dysfunction) |
| VIP | M=(cesarean delivery OR cesarean section OR caesarean delivery OR caesarean section OR C-section OR delivery OR delivery mode OR mode of delivery) AND M=(ADHD OR attention deficit hyperactivity disorder OR attention deficit disorder OR hyperkinetic disorder OR hyperkinetic syndrome OR childhood hyperactivity OR hyperactive child syndrome OR minimal brain dysfunction) |

**Original version of the search strategy**

| Database | Search strategy |
| --- | --- |
| PubMed | (mode of delivery[Title/Abstract] OR method of delivery[Title/Abstract] OR delivery[Title/Abstract] OR birth delivery[Title/Abstract] OR caesarean birth[Title/Abstract] OR cesarean[Title/Abstract] OR cesarean section[Title/Abstract] OR Caesarean section[Title/Abstract] OR cesarean delivery[Title/Abstract]) AND ((ADHD[Title/Abstract] OR adhd[Title/Abstract] OR attention deficit hyperactivity disorder[Title/Abstract] OR child hyperkinetic syndrome[Title/Abstract] OR childhood hyperkinetic syndrome[Title/Abstract] OR infantile hyperkinetic syndrome[Title/Abstract])) |
| the Cochrane Library | mode of delivery OR method of delivery OR delivery OR birth delivery OR caesarean birth OR cesarean OR cesarean section OR Caesarean section OR cesarean delivery:ti,ab,kw ADHD OR adhd OR attention deficit hyperactivity disorder OR child hyperkinetic syndrome OR childhood hyperkinetic syndrome OR infantile hyperkinetic syndrome:ti,ab,kw |
| Web of Science | TS=(mode of delivery OR method of delivery OR delivery OR birth delivery OR caesarean birth OR cesarean OR cesarean section OR Caesarean section OR cesarean delivery ) AND TS= (ADHD OR adhd OR attention deficit hyperactivity disorder OR child hyperkinetic syndrome OR childhood hyperkinetic syndrome OR infantile hyperkinetic syndrome) |
| CNKI | SU=('剖宫产'+'cesarean delivery'+'cesarean section'+'剖腹产'+'剖腹产术'+'剖宫产术'+'分娩方式') AND SU=('ADHD'+'儿童多动症'+'adhd'+'attention deficit hyperactivity disorder'+'child hyperkinetic syndrome'+'childhood hyperkinetic syndrome'+'infantile hyperkinetic syndrome'+'注意力缺陷多动症'+'多动综合症'+'注意缺陷多动障碍'+'多动症'+'注意缺陷与多动障碍'+'多动综合征'+'注意力缺陷障碍'+'注意力缺陷伴多动障碍'+'注意缺陷-多动障碍'+'多动性障碍'+'儿童多动综合症'+'儿童多动综合征'+'多动儿童'+'儿童注意力缺陷多动障碍'+'儿童注意缺陷多动障碍') |
| Wanfang | 主题:(剖宫产 or cesarean delivery or cesarean section or 剖腹产 or 剖腹产术 or 剖宫产术or 分娩or 分娩方式) and 主题:(ADHD or 儿童多动症 or adhd or attention deficit hyperactivity disorder or child hyperkinetic syndrome or childhood hyperkinetic syndrome or infantile hyperkinetic syndrome or 注意力缺陷多动症 or 多动综合症 or 注意缺陷多动障碍 or 多动症 or 注意缺陷与多动障碍 or 多动综合征 or 注意力缺陷障碍 or 注意力缺陷伴多动障碍 or 注意缺陷-多动障碍 or 多动性障碍 or 儿童多动综合症 or 儿童多动综合征 or 多动儿童 or 儿童注意力缺陷多动障碍 or 儿童注意缺陷多动障碍) |
| VIP | M=(剖宫产 or cesarean delivery or cesarean section or 剖腹产 or 剖腹产术 or 分娩 or 分娩方式 ) AND M=(ADHD or 儿童多动症 or adhd or attention deficit hyperactivity disorder or child hyperkinetic syndrome or childhood hyperkinetic syndrome or infantile hyperkinetic syndrome or 注意力缺陷多动症 or 多动综合症 or 注意缺陷多动障碍 or 多动症 or 注意缺陷与多动障碍 or 多动综合征 or 注意力缺陷障碍 or 注意力缺陷伴多动障碍 or 注意缺陷-多动障碍 or 多动性障碍 or 儿童多动综合症 or 儿童多动综合征 or 多动儿童 or 儿童注意力缺陷多动障碍 or 儿童注意缺陷多动障碍) |
