# Supplementary material for: The relationship between mode of delivery and Attention Deficit Hyperactivity Disorder: a meta-analysis and systematic review
Source: PeerJ. 2026 Jan 16;14:e20603. doi: 10.7717/peerj.20603 (PMC12814906; doi:10.7717/peerj.20603)
Supplement: Supplemental Information 4 — Quality assessment results for case-control studies included in the meta-analysis using the Newcastle-Ottawa Quality Assessment Scale (NOS) checklist. The NOS evaluates study quality based on selection of study groups, comparability of groups, and ascertainment of exposure. [file peerj-14-20603-s004.docx]

**Supplementary Table 3.** **Quality assessment of case-control studies included.**

| Author, year,  Study (Observational) | **Selection (Out of 4)** | | | | **Comparability**  **(Out of 2)** | **Outcomes (Out of 3)** | | | **Total**  **(Out of 9)** |
| --- | --- | --- | --- | --- | --- | --- | --- | --- | --- |
|  | Adequate case definition | Representativeness of the cases | Selection of controls | Definition of controls |  | Ascertainment of exposure | Same method of ascertainment for cases and controls | Non-response rate |  |
| Amiri 2012 | 1 | 1 | 1 | 1 | 1 | 1 | 1 | 0 | 7 |
| Halmoy 2011 | 1 | 1 | 1 | 1 | 2 | 1 | 1 | 1 | 9 |
| Pohlabeln 2017 | 1 | 1 | 1 | 1 | 1 | 1 | 1 | 1 | 8 |
| Silva 2013 | 1 | 1 | 1 | 1 | 1 | 1 | 1 | 1 | 8 |
| Sucksdorff 2018 | 1 | 1 | 1 | 1 | 1 | 1 | 1 | 1 | 8 |
| Zhou 2024 | 1 | 1 | 1 | 1 | 1 | 1 | 1 | 1 | 8 |

The observational studies were assessed by the Newcastle-Ottawa Quality Assessment Scale (NOS) checklist of case-control studies.
