# Supplementary material for: The relationship between mode of delivery and Attention Deficit Hyperactivity Disorder: a meta-analysis and systematic review
Source: PeerJ. 2026 Jan 16;14:e20603. doi: 10.7717/peerj.20603 (PMC12814906; doi:10.7717/peerj.20603)
Supplement: Supplemental Information 5 — Quality assessment results for cohort studies included in the meta-analysis using the Newcastle-Ottawa Quality Assessment Scale (NOS) checklist. The NOS evaluates study quality based on selection of study groups, comparability of groups, and assessment of outcomes. [file peerj-14-20603-s005.docx]

**Supplementary Table 2.** **Quality assessment of cohort studies included.**

| Author, year,  Study (Observational) | **Selection (Out of 4)** | | | | **Comparability**  **(Out of 2)** | **Outcomes (Out of 3)** | | | **Total**  **(Out of 9)** |
| --- | --- | --- | --- | --- | --- | --- | --- | --- | --- |
|  | Representativeness of exposed cohort | Selection of non exposed cohort | Ascertainment  of exposure | Outcome not present at the start of the study |  | Assessment of outcomes | Length of follow-up | Adequacy of follow up of cohorts |  |
| Axelsson 2018 | 1 | 0 | 1 | 1 | 1 | 1 | 1 | 1 | 7 |
| Chen 2022 | 1 | 1 | 1 | 1 | 2 | 1 | 1 | 0 | 7 |
| Curran 2015 | 1 | 1 | 1 | 1 | 2 | 1 | 1 | 1 | 9 |
| Curran 2016 | 1 | 1 | 1 | 1 | 2 | 1 | 1 | 1 | 9 |
| Jin 2013 | 1 | 1 | 1 | 1 | 1 | 1 | 0 | 1 | 7 |
| Murray 2015 | 1 | 1 | 1 | 1 | 1 | 1 | 1 | 1 | 7 |
| Njotto 2023 | 1 | 1 | 1 | 1 | 1 | 1 | 1 | 1 | 8 |
| Schwenke 2018 | 1 | 1 | 1 | 1 | 1 | 0 | 1 | 1 | 7 |

The observational studies were assessed by the Newcastle-Ottawa Quality Assessment Scale (NOS) checklist of cohort studies.
