# Supplementary figures and images for: The relationship between mode of delivery and Attention Deficit Hyperactivity Disorder: a meta-analysis and systematic review
Source: PeerJ. 2026 Jan 16;14:e20603. doi: 10.7717/peerj.20603 (PMC12814906; doi:10.7717/peerj.20603)

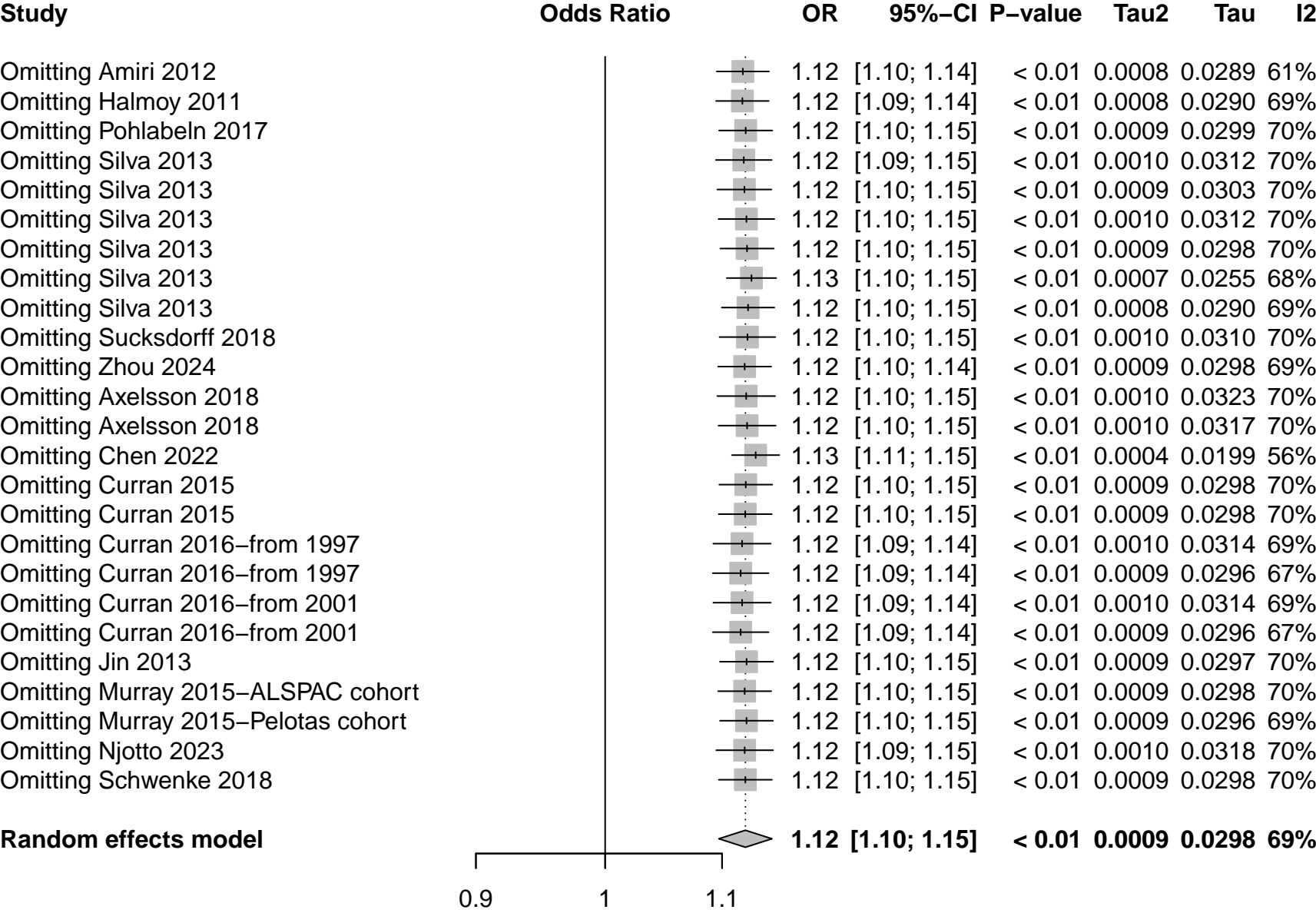

Supplement: Supplemental Information 6 — Results of sensitivity analysis examining the robustness of pooled estimates by systematically excluding each study one at a time. The table shows odds ratios, confidence intervals, p-values, and heterogeneity statistics (I2) for each omission scenario to assess the influence of individual studies on overall results. [file peerj-14-20603-s006.pdf]
