# Supplementary material for: The relationship between mode of delivery and Attention Deficit Hyperactivity Disorder: a meta-analysis and systematic review
Source: PeerJ. 2026 Jan 16;14:e20603. doi: 10.7717/peerj.20603 (PMC12814906; doi:10.7717/peerj.20603)
Supplement: Supplemental Information 7 [file peerj-14-20603-s007.docx]

**English-Language Codebook for Chinese Search Terms**

**Table S1 Supplementary Material: English Translation Codebook for Chinese Database Search Terms**

1. **ADHD-Related Terms (注意力缺陷多动障碍相关术语)**

| **Chinese Term** | **Pinyin** | **English Translation** |
| --- | --- | --- |
| ADHD | ADHD | Attention Deficit Hyperactivity Disorder |
| 儿童多动症 | értóng duōdòng zhèng | Childhood hyperactivity disorder |
| adhd | adhd | Attention deficit hyperactivity disorder (lowercase) |
| 注意力缺陷多动症 | zhùyìlì quēxiàn duōdòng zhèng | Attention deficit hyperactivity disorder |
| 多动综合症 | duōdòng zònghé zhèng | Hyperkinetic syndrome |
| 注意缺陷多动障碍 | zhùyì quēxiàn duōdòng zhàng'ài | Attention deficit hyperactivity disorder |
| 多动症 | duōdòng zhèng | Hyperactivity disorder |
| 注意缺陷与多动障碍 | zhùyì quēxiàn yǔ duōdòng zhàng'ài | Attention deficit and hyperactivity disorder |
| 多动综合征 | duōdòng zònghé zhēng | Hyperkinetic syndrome |
| 注意力缺陷障碍 | zhùyìlì quēxiàn zhàng'ài | Attention deficit disorder |
| 注意力缺陷伴多动障碍 | zhùyìlì quēxiàn bàn duōdòng zhàng'ài | Attention deficit disorder with hyperactivity |
| 注意缺陷-多动障碍 | zhùyì quēxiàn-duōdòng zhàng'ài | Attention deficit-hyperactivity disorder |
| 多动性障碍 | duōdòng xìng zhàng'ài | Hyperactivity disorder |
| 儿童多动综合症 | értóng duōdòng zònghé zhèng | Childhood hyperkinetic syndrome |
| 儿童多动综合征 | értóng duōdòng zònghé zhēng | Childhood hyperkinetic syndrome |
| 多动儿童 | duōdòng értóng | Hyperactive children |
| 儿童注意力缺陷多动障碍 | értóng zhùyìlì quēxiàn duōdòng zhàng'ài | Childhood attention deficit hyperactivity disorder |
| 儿童注意缺陷多动障碍 | értóng zhùyì quēxiàn duōdòng zhàng'ài | Childhood attention deficit hyperactivity disorder |

1. **Delivery Mode-Related Terms (分娩方式相关术语)**

| **Chinese Term** | **Pinyin** | **English Translation** |
| --- | --- | --- |
| 剖宫产 | pōu gōng chǎn | Cesarean section |
| 剖腹产 | pōu fù chǎn | Cesarean delivery |
| 剖腹产术 | pōu fù chǎn shù | Cesarean section procedure |
| 剖宫产术 | pōu gōng chǎn shù | Cesarean section surgery |
| 分娩方式 | fēn miǎn fāng shì | Mode of delivery / Delivery method |
| 分娩 | fēn miǎn | Delivery / Childbirth |

1. **Database Search Strategy Notes:**
   1. **CNKI (China National Knowledge Infrastructure)**: Used subject heading search (SU=) combining ADHD and delivery mode terms
   2. **Wanfang Database**: Used subject search (主题:) with OR operators between synonymous terms
   3. **VIP Database**: Used main subject search (M=) with AND operators between concept groups
